# Enhancing exercise performance and recovery through sodium bicarbonate supplementation: introducing the ingestion recovery framework

**DOI:** 10.1007/s00421-024-05578-0

**Published:** 2024-08-23

**Authors:** William H. Gurton, David G. King, Mayur K. Ranchordas, Jason C. Siegler, Lewis A. Gough

**Affiliations:** 1 Sport & Physical Activity Research Centre, Sheffield Hallam University, Sheffield, UK; 2 School of Energy, Geoscience, Infrastructure and Society, Institute for Life and Earth Sciences, Heriot Watt University, Edinburgh, Scotland, UK; 3 Advanced Wellbeing Research Centre, Sheffield, UK; 4 College of Health Solutions, Arizona State University, Phoenix, AZ USA; 5 Human Performance and Health Research Group, Centre for Life & Sport Sciences (CLaSS), Birmingham City University, Birmingham, UK

**Keywords:** Acid–base balance, Recovery, Repeated bout exercise, Supplements

## Abstract

Sodium bicarbonate (SB) supplementation is an ergogenic strategy for athletes competing in high-intensity exercise, but the efficacy of SB for accelerating recovery from exercise and thus improving performance during repeated bouts of exercise is not fully understood. In a similar fashion to using SB as a pre-exercise buffer, it is possible accelerated restoration of blood pH and bicarbonate following an exercise bout mechanistically underpins the use of SB as a recovery aid. Physiological mechanisms contributing to beneficial effects for SB during repeated bout exercise could be more far-reaching however, as alterations in strong ion difference (SID) and attenuated cellular stress response might also contribute to accelerated recovery from exercise. From inspection of existing literature, ingestion of 0.3 g kg^−1^ body mass SB ~60–90 min pre-exercise seems to be the most common dosage strategy, but there is evidence emerging for the potential application of post-exercise supplementation timing, gradual SB doses throughout a competition day, or even ingestion during exercise. Based on this review of literature, an SB ingestion recovery framework is proposed to guide athletes and practitioners on the use of SB to enhance performance for multiple bouts of exercise.

## Introduction

Sodium bicarbonate (SB) supplementation prior to exercise is an ergogenic strategy for improving athletic performance during single and repeated bouts of high-intensity exercise (Carr et al. 2011a; de Oliveira et al. 2022). Unlike many commercially available sport supplements, there is empirical evidence supporting the efficacy of SB, and thus, it was recommended for use by athletes in the 2018 International Olympics Committee (IOC) consensus statement (Maughan et al. 2018). Ergogenic benefits following pre-exercise SB ingestion have traditionally been linked to the assumption that accumulation of hydrogen cations (H^+^) in the cytosol of muscle fibres, which by definition computes to a decrease in intracellular pH, may contribute towards skeletal muscle fatigue (Fitts 2016). Whilst the deleterious effects of H^+^ accumulation on skeletal muscle function have been debated (Westerblad 2016), it is proposed that offsetting declining intramuscular pH during intense exercise prevents the inhibition of metabolic processes required to generate adenosine triphosphate (ATP) (Spriet et al. 1989; Messonnier et al. 2007) and sustain rates of contractile cycling (Debold et al. 2008).

Ingesting SB augments the body’s extracellular buffering capacity by increasing blood bicarbonate (HCO_3_^−^) concentration, subsequently allowing for greater efflux of H^+^ from contracting muscles and thus protecting against biochemical disturbances to intramuscular acid–base balance (Hollidge-Horvat et al. 2000; Bishop et al. 2004). SB ingestion has also been shown to elicit changes in intra- and extracellular distribution of ions [e.g., sodium (Na^+^), potassium (K^+^), calcium (Ca^2+^), and chloride (Cl^−^)] that may preserve muscle excitability during intense exercise (Cairns and Lindinger 2008; Kent-Braun et al. 2012). Since there is likely no singular mechanism underpinning the performance enhancing effects of SB ingestion, it is important researchers adopt a multifaceted perspective when studying physiological systems that may contribute.

Most independent placebo-controlled studies and review articles have focused on physiological responses and ergogenic benefits for SB from its context as a pre-exercise extracellular buffer. A brief inspection of scientific literature reveals that a considerable number of research studies (~200) have been conducted investigating the effect of SB ingestion on exercise performance. Work dates back as far as 1930 when Dennig et al. (1931) first reported pre-exercise SB improved accumulated oxygen debt during 15 min steady-state running. Throughout the past decade, numerous meta-analyses have shown pre-exercise SB ingestion to elicit moderate performance benefits, with the greatest improvements thought to exist during exercise tasks lasting between 45 s and 10 min (Carr et al. 2011a; Peart et al. 2012; Hadzic et al. 2019; de Oliveira et al. 2022). Compared to this widely accepted use of SB ingestion as a pre-exercise ergogenic aid, less focus has been given to the potential application of SB for improving recovery between two or more exercise bouts. From our experience of talking to members of the scientific community and applied practitioners, it is evident that SB is still viewed as a pre-exercise extracellular buffering aid. As scientific literature becomes increasingly overwhelmed by research studies examining the effect of SB on exercise performance, it is believed that shifting the narrative more towards studies examining whether SB is able to accelerate recovery from exercise will provide researchers with a fresh perspective and maximise practical implications for athletes.

One challenge, however, is precisely defining what “recovery from exercise” means from a physiological viewpoint. In short, it can be termed as the amount of time between finishing an initial exercise bout and the subsequent restoration of physiological systems towards baseline levels (Luttrell and Halliwill 2015). That being said, the temporal definition of “recovery” varies depending on the physiological system or pathway being studied. In light of these different interpretations of “recovery from exercise”, it is important to provide a clear explanation of how SB may accelerate recovery between two or more bouts of exercise. In the context of SB ingestion and recovery, numerous studies have showed SB ingestion prior to an initial exercise bout elevates blood pH and [HCO_3_^−^] during recovery periods lasting 15–40 min (Siegler et al. 2008; Pruscino et al. 2008; Gough et al. 2018). Considering the potential role of extracellular buffering capacity for protecting against declining intramuscular pH and offsetting skeletal muscle fatigue (Fitts 2016), accelerating restoration of blood acid–base balance prior to subsequent exercise bouts might be crucial to maximising performance. In a similar fashion, there is a small body of literature suggesting that pre-exercise SB ingestion may accelerate the rate at which strong ion difference (SID) recovers to baseline levels after exercise (Sostaric et al. 2006; Gough et al. 2019a, b). These ionic changes are thought to have important implications for preserving muscle excitability (Cairns and Lindinger 2008), which may contribute to force generating capacity. From a longer term sense on a “recovery” continuum, a series of studies investigated whether SB was able to attenuate cellular stress responses and oxidative stress up to 24 h following exercise (Peart et al. 2011, 2013a, 2016). The authors reported that SB ingestion alleviates production of reactive oxygen species (ROS) (Peart et al. 2011), and this might reduce oxidative stress and improve skeletal muscle function (Powers and Jackson 2008).

**Fig. 1:**
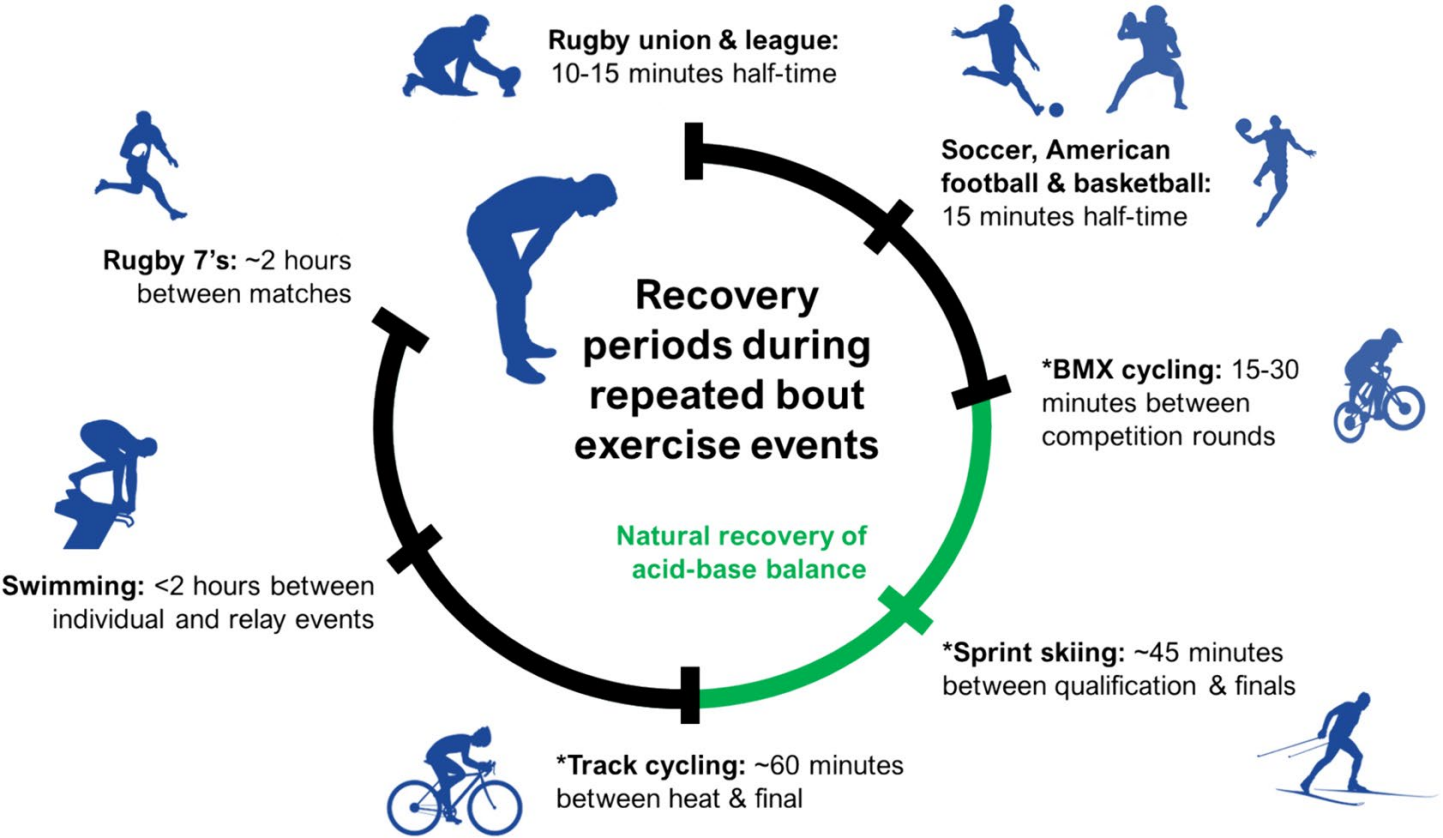
Schematic representation of sporting disciplines that may benefit from using SB as a recovery aid. Approximate timescales between repeated bouts of exercise presented as a chronological sequence. Green section denotes estimated duration required for blood acid–base balance status to recover to baseline following high-intensity exercise. * Denotes the timeframe that a single SB dose has improved recovery and performance in studies to date

Although further research is required to better understand how SB ingestion influences these physiological mechanisms and consequently accelerates “recovery from exercise”, there is a strong ‘real-world’ rationale for the use of SB as a recovery aid. Assuming that SB restores homeostasis of physiological pathways that contribute to skeletal muscle fatigue during an initial exercise bout, then logically SB ingestion would be an ergogenic strategy for athletes required to perform multiple exercise bouts on the same day (Fig. 1). For instance, sporting disciplines such as swimming and BMX/track cycling that see athletes take part in heat/qualification rounds and finals throughout a single competition day (Mero et al. 2013; Peinado et al. 2019). There might also be scope for using SB as a recovery aid during sports such as rugby sevens and cross-country skiing, with both characterised by excessive H^+^ accumulation during exercise and featuring short recovery periods between repeated exercise bouts (Ross et al. 2014; Losnegard 2019). Application of SB during these sports is yet to receive scientific interest, but it is hoped that this review will educate readers on the potential beneficial effects of SB ingestion as a recovery aid and help conceptualise future scientific work across a variety of sporting disciplines.

In light of the potential benefits of SB ingestion for accelerating “recovery from exercise”, this review will first discuss how SB ingestion could influence recovery of three pertinent physiological systems/pathways: (1) acid–base balance, (2) SID, and (3) cellular stress responses and oxidative stress. From an applied perspective, it is crucial to produce clear recommendations of how to use SB for athletes competing in multiple exercise bouts on the same day. As such, second, we will outline the significance of dosage, ingestion strategy and timing for optimising blood buffering capacity and repeated bout exercise performance. Additionally, this review will provide a comprehensive evaluation of findings from placebo-controlled research studies investigating the effect of SB ingestion on repeated bout exercise performance. For the purpose of this review, we are defining repeated bout exercise performance as “two or more exercise events performed on the same day separated by at least 10 min recovery”. This temporal pattern of “recovery” was based off previous studies examining the effect of SB on acid–base balance recovery, whereby ~10 min following an initial exercise bout is when blood pH and [HCO_3_^−^] started their recovery towards baseline (Verbitsky et al. 1997; Siegler et al. 2008). Whilst outside the scope of this review, potential ergogenic benefits of SB ingestion during repeated and intermittent exercise comprising shorter work/recovery ratios have been discussed elsewhere (Lopes-Silva et al. 2019; Grgic 2022). Finally, based on the existing literature, an SB ingestion framework will be presented for athletes and coaches that wish to incorporate the supplement into their nutritional regimes.

## Proposed mechanisms for accelerating recovery from exercise

In a similar fashion to studies examining physiological mechanisms underpinning the use of SB as a pre-exercise buffer (Siegler et al. 2016), greatest attention has been given to effect of SB ingestion on the recovery of blood acid–base balance after exercise (Pruscino et al. 2008; Gough et al. 2019a, b). Early work by Costill et al. (1984) observed that compared to a placebo, ingesting SB prior to 5 × 1 min cycling bouts increased blood pH [~0.05 arbitrary units (au)] and [HCO_3_^−^] (~4 mmol L^−1^) after 30 min recovery (both *p*<0.05). In agreement with these findings, studies have shown SB ingested pre-exercise to elevate blood pH and [HCO_3_^−^] during recovery periods ranging between 15 and 40 min post-exercise (Pruscino et al. 2008; Siegler et al. 2008; Gough et al. 2018). Additionally, higher blood pH and [HCO_3_^−^] have been reported following 75–90 min recovery when SB was ingested after an initial exercise bout (Gough et al. 2017a, 2019a, b).

**Fig. 2:**
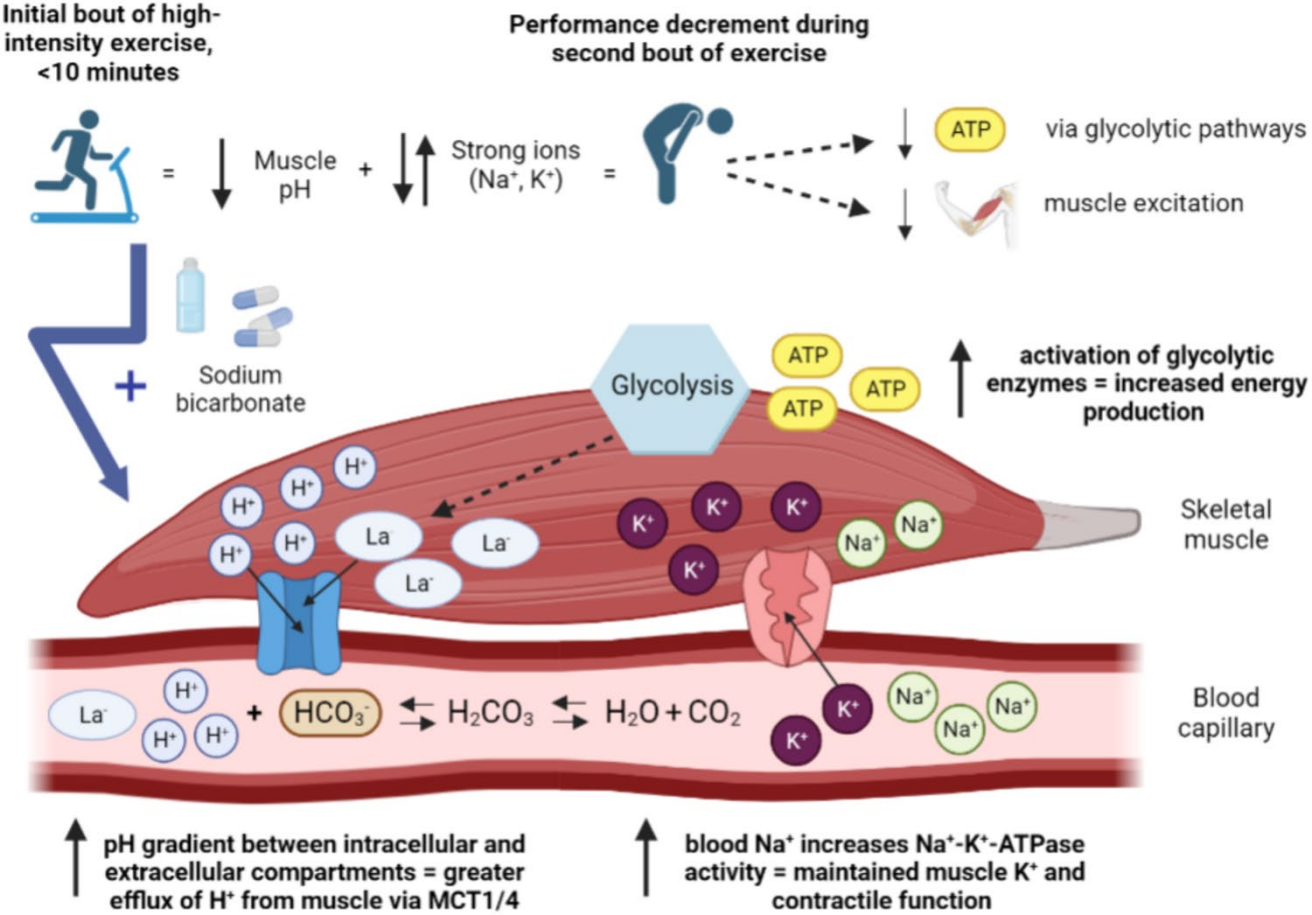
Schematic outlining a focused viewpoint of how changes in acid–base balance and strong ions mechanistically underpin performance benefits for repeated bouts of high-intensity exercise following SB ingestion. Top section: exercise-induced disruptions to acid–base balance and strong ion difference. Bottom section: physiological changes (shown in bold) following SB ingestion that contribute towards improvements in performance. (Schematic created in BioRender.com)

It should be noted that the aforementioned studies all focused on changes occurring in extracellular compartments, but evidence from early animal model work suggests that similar responses may occur within intramuscular compartments following alkalosis (Spriet et al. 1985, 1986; Bishop et al. 2004). As such, is it reasonable to assume that SB ingestion would also have raised intramuscular acid–base balance prior to subsequent exercise, which could protect against the inhibition of key glycolytic enzymes (i.e., glycogen phosphorylase) during subsequent exercise, thus increasing the rate of glycolytic flux and ATP production (Fig. 2) (Messonnier et al. 2007; Fitts 2016). This could be particularly true when only a limited recovery window is available and athletes without SB ingestion would commence the second bout in a fatigued state and their acid–base balance would be below baseline levels.

Whilst current findings seem to suggest that ingestion of SB is an effective strategy for elevating blood acid–base balance status between repeated bouts of exercise, it might be misleading to base conclusions regarding its efficacy from studies with only a few blood sampling points during recovery. Robergs et al. (2005) comprehensively examined short- (i.e., 2 min sampling points for the first 20 min) and long-phase (i.e., 4 min sampling points for the final 40 min) blood pH and HCO_3_^−^ recovery pharmacokinetics throughout a 60 min recovery period after pre-exercise SB ingestion. These researchers demonstrated that SB increased blood pH and [HCO_3_^−^] up to 60 min after time-to-exhaustion (TTE) cycling compared to a placebo. Interestingly, they also reported that blood pH recovery on monoexponential slopes (~0.6) and half-time constants (*t*_0.5_ = ~12-min) were not different between SB and placebo. In short, these results from Robergs et al. (2005) suggest that the higher blood pH and [HCO_3_^−^] reported following SB ingestion during recovery from intense exercise might not be related to accelerated blood acid–base balance recovery pharmacokinetics per se, but instead to reduced acid–base balance perturbations during the initial exercise bout (Bishop et al. 2004).

Since most studies report significantly higher blood pH and [HCO_3_^−^] immediately post-exercise after SB supplementation, it is possible that dampened metabolic perturbation during the initial exercise bout contributes to acid–base balance recovering back to baseline levels faster (i.e., recovery starts from a higher level). However, studies that have adopted a post-exercise ingestion strategy to ensure SB supplementation occurred from a state of metabolic acidosis consistent with placebo suggest SB does accelerate recovery (Gough et al. 2019a, b; Gurton et al. 2021a). Post-exercise ingestion of SB elevated blood acid–base balance prior to and/or after a second exercise bout compared to a placebo, but the lack of blood sampling points during recovery (i.e., only measured at the start and end) meant that it was difficult to evaluate the efficacy of SB for accelerating pH and HCO_3_^−^ recovery pharmacokinetics. Future studies should replicate the methodology used by Robergs et al. (2005) to examine the effect of post-exercise SB on acid–base balance recovery pharmacokinetics (i.e., monoexponential slopes, half-time constants) to unpick the mechanism further.

Another mechanism that may underpin the application of SB ingestion as a recovery aid are alterations to SID (Sostaric et al. 2006; Gough et al. 2019a, b). Disturbances to the SID within skeletal muscle and surrounding interstitium during intense exercise contribute towards muscle fatigue by depressing maximal Na^+^,K^+^-ATPase activity, subsequently impairing cell membrane excitability (Fraser et al. 2002; Sostaric et al. 2006). SB ingestion may increase influx of K^+^ into muscles and attenuate exercise-induced release of K^+^ back into extracellular mediums (Siegler and Gleadall-Siddall 2010). Combined with increased muscular Cl^−^ uptake and plasma [Na^+^] following SB (Fig. 2) (Siegler and Hirscher 2009; Gough et al. 2017b), it is possible that SB upregulates Na^+^/K^+^-ATPase and Na^+^/K^+^/2Cl^−^-ATPase activity to limit depolarization and preserve excitation–contraction coupling (Allen et al. 2008). In vitro experimental models have demonstrated that stimulating Na^+^–K^+^-ATPase activity delays muscle fatigability and accelerates subsequent recovery (Clausen and Everts 1991; Clausen et al. 1993), and therefore, SB mediated changes in SID during initial fatiguing exercise tasks could also be beneficial to repeated exercise performance. Indeed, Sostaric et al. (2006) examined the effect of SB on SID during exhaustive dynamic forearm exercise and found that plasma [Na^+^] and muscle Cl^−^ uptake were higher for SB compared with a control. Somewhat unexpectedly, SB increased release of K^+^ from muscles into plasma at fatigue (~49% higher compared with control; *p*<0.05). This finding could partly be attributed to greater work completed during the exhaustive exercise task (25% greater than placebo; *p*<0.05). On the other hand, Sostaric et al. (2006) also observed that SB ingestion elevated uptake of K^+^ into the muscles compared with a placebo throughout a 10-min recovery (~17% higher), which the authors attributed to the higher K^+^ release at fatigue following SB ingestion and the opening of some K^+^ channels owing to augmented Na^+^, K^+^-ATPase (and possibly Na^+^/K^+^/2Cl^−^-ATPase) activity. However, as Sostaric et al. (2006) investigated a finger flexion exercise task, it remained unclear for years whether similar changes would be observed for whole-body repeated bout exercise.

More recently, in a series of investigations, Gough et al. (2018, 2019a, b) explored the effect of SB on SID during ecologically valid exercise protocols [i.e., 4-km time trials (TT), boxing simulation]. Studying SID instead of individual changes in SID allows the collective assessment of the balance of fully dissociated cations and anions in intra- and extracellular fluid (Stewart 1983). In two similar studies, Gough et al. (2018, 2019a) observed that SB accelerated restoration of arterialised (finger) blood SID during 40-min recovery periods after 4-km cycling TT in normobaric hypoxic conditions (~5% greater than placebo; *p*<0.05). Expanding upon these findings, Gough et al. (2019b) demonstrated higher blood SID (~10% greater than placebo; *p*<0.001) after 75 min recovery following a TTE running/boxing simulation exercise task. These researchers attributed accelerated recovery of SID to elevated blood [Na^+^] concomitant with reductions in blood [K^+^] and [Cl^−^] for SB compared to the placebo, consequently inferring increased uptake of K^+^ and Cl^−^ into the muscles to preserve excitation–contraction coupling and action potentials (Fig. 2). Whilst both studies employed a second bout of exercise and reported performance benefits for SB, only Gough et al. (2019b) showed SID to remain elevated for SB compared to placebo, in turn raising questions about whether collective changes in SID significantly influence repeated bout exercise performance. Interestingly, Gough et al. (2018) observed that blood [K^+^] was higher for SB after their second 4-km cycling TT, reinforcing previous research that changes in [K^+^] may play the most important role of the strong ions for sustaining muscle excitability (Cairns and Lindinger 2008).

Since muscle force is substantially depressed by reduced intracellular-to-extracellular [K^+^] ratio (Cairns et al. 1995; Nielsen et al. 1998), it is likely lower plasma [K^+^] after SB ingestion contributes to maintaining a high intracellular-to-extracellular [K^+^] ratio (Sostaric et al. 2006). Of note, however, is that the results from Gough et al. (2018, 2019a, b) only reveal changes occurring in extracellular compartments. This does not directly confirm SB protected against ionic disturbances within contracting muscle, leaving it as an avenue for future research to explore.

Finally, there is a small body of scientific literature examining the effect of pre-exercise SB on physiological stress responses during recovery from high-intensity exercise (Peart et al. 2011, 2013a, b, 2016). Researchers have focused on the relationship between pre-exercise alkalosis and cellular stress responses [i.e., intracellular heat shock protein 72 (HSP72), oxidative stress]. Briefly, expression of intramuscular HSP72 increases after high-intensity exercise to protect against stress-induced apoptosis (Mosser et al. 1997) due to production of reactive oxygen species (ROS) via cellular respiration (Taylor et al. 2011). Since in vitro experimental models have demonstrated that HSP72 response is also linked to an intramuscular acidosis (Gapen and Moseley 1995), it was theorized that greater efflux of H^+^ from cells following SB may dampen HSP72 expression after intense exercise. Peart et al. (2011) demonstrated that SB decreased HSP72 response after “all-out” anaerobic cycling (~40% lower than placebo; *p*=0.013). They also reported reduced post-exercise lipid peroxidation [via plasma thiobarbituric acid reactive substances (TBARS)] following SB. Attenuated HSP72 for SB could be attributed to reduced oxidative stress, as greater efflux of H^+^ from intramuscular compartments alleviates production of ROS during cellular respiration that would otherwise impair skeletal muscle function (Powers and Jackson 2008). Peart et al. (2011) acknowledged that their results should be interpreted with caution however, since TBARS is not exclusively related to lipid peroxidation, meaning that it might overestimate oxidative stress (Koster et al. 1985; Powers et al. 2010). Across two follow-up studies, Peart et al. (2013a, b) examined the effect of SB ingestion on exercise-specific markers of oxidative stress, including total antioxidant capacity, total glutathione (TGSH), and oxidised glutathione (GSSG). SB attenuated HSP72 expression and TBARS concentration after repeated sprint cycling, although no differences were reported between treatments for either total antioxidant capacity or the ratio of GSSG/TSGH. In light of these results, Peart et al. (2013a, b) concluded that the maintenance of intracellular acid–base balance following SB ingestion is likely the primary factor contributing to reductions in HSP72 expression, as opposed to changes in ROS generation.

Interestingly, recent findings from Thomas et al. (2023) demonstrated that SB decreased oxidative stress (24 h post-exercise protein carbonylation ~30% lower than placebo; *p*<0.05) after performing 3 × 30 s Wingate cycling tests. Additional research is required to further understand the effect of SB ingestion on post-exercise oxidative stress, but regardless of the underlying physiological mechanisms, dampening HSP72 responses after an initial exercise task could have practical implications for recovery as theoretically athletes would experience less physiological stress during subsequent exercise bouts.

Importantly, depending on the specific situation, the prior results suggest that SB could be used to reduce exercise stress (i.e., during competition) or enhance physiological adaptations from training. This hypothesis was studied during work by Peart et al. (2016) that investigated whether SB mediated changes in post-exercise HSP72 expression influenced cellular stress responses following a second bout of high-intensity exercise (bout 1: 10 × 15 s sprints; bout 2:90 min intermittent sprint cycling). HSP72 expression was reduced for SB following the first exercise bout (~25% lower than placebo; *p*=0.05), but was similar between treatments after the second bout. Whilst these results suggest that improvements in intramuscular acid–base balance do not reduce cellular stress responses during repeated exercise bouts, it is possible that the magnitude of HSP72 expression following their initial exercise task was not high enough to influence physiological stress responses during subsequent exercise (Taylor et al. 2011). Additional studies are required to examine the effect of SB on post-exercise oxidative stress, including ingestion during heavy workloads (i.e., especially in competition) that induce substantial HSP72 expression.

## Dosage, ingestion strategy, and timing

Researchers examining the effect of SB on recovery from exercise have typically chosen 0.2–0.3 g kg^−1^ BM doses (Costill et al. 1984; Pierce et al. 1992; Gough et al. 2017a). Similarities likely originate from early work by McNaughton (1992) that proposed 0.3 g kg^−1^ BM SB ingested pre-exercise induced a sufficient blood alkalosis ([HCO_3_^−^]: ~31 mmol L^−1^), without causing severe gastrointestinal (GI) discomfort unlike their 0.4–0.5 g kg^−1^ BM doses. Since more contemporary research has suggested that 0.2 g kg^−1^ BM SB elicits substantial changes in [HCO_3_^−^] and alleviates GI discomfort compared to 0.3 g kg^−1^ BM (Gough et al. 2018; Gurton et al. 2020), it could be an attractive dose for athletes known to suffer from side effects (Gough et al. 2018; Gurton et al. 2020). The composition of SB is ~27% Na^+^ (assuming 1 mol SB is 84 g mol^−1^), meaning that a 70 kg athlete ingesting 0.4 g kg^−1^ BM SB would consume ~7500 mg of Na^+^, which exceeds the recommended upper limit of 2300 mg for daily intake (Bibbins-Domingo 2014). As such, athletes regularly consuming SB could be at higher risk of developing cardiovascular complications (i.e., hypertension) linked to excessive dietary Na^+^ intake (Strazzullo et al. 2009). Whilst outside the scope of this review, future studies should examine the impact of chronic SB supplementation on long-term health outcomes.

Traditionally, most placebo-controlled studies have opted to administer SB in solution or capsules (gelatine/vegetarian/enteric-coated) (Gurton et al. 2023a), but within recent years, topically applied lotion (Gurton et al. 2023b) and minitabs (Gough and Sparks 2024) have also emerged within the literature. Regardless of personal preferences for dose and supplement timing, solution and capsule administration approaches are considered best practice (de Oliveira et al. 2022). A key determinant of the ingestion method used by researchers and/or practitioners is the resulting GI discomfort. In a study examining eight SB supplementation approaches, Carr et al. (2011b) reported that solution ingestion protocols cause more severe side effects compared to capsules. These authors concluded that coingestion of SB alongside a carbohydrate meal (~1.5 g kg^−1^ BM) further reduced the occurrence of GI discomfort, although does not guarantee side effects will be eliminated completely (Carr et al. 2011b). It has also been suggested that splitting SB into multiple doses prior to exercise can alleviate GI discomfort, whilst still inducing a sufficient pre-exercise alkalosis (HCO_3_^−^ ~32 mmol L^−1^; pH ~7.50 au; Saunders et al. 2014). One drawback, however, is that a split-dosage approach may not be effective if ingestion of SB is needed after an initial exercise bout, as there would likely be insufficient time available to see elevated acid base balance recovery pharmacokinetics.

Recently, two novel forms of SB administration, topically applied lotion (PR Lotion; Momentous) and minitabs with a hydrogel carbohydrate solution (Maurten, Sweden) have emerged within the literature. To date, equivocal findings exist for topical SB (McKay et al. 2020; Gibson et al. 2023), but Gurton et al. (2023b) observed improvements in 8 × 25 m repeated running sprint times for topical SB (~2%; *p*=0.036) without any GI discomfort. On the other hand, Gough and Sparks (2024) reported that aggregated GI discomfort (i.e., sum of all GI symptoms) was reduced for a hydrogel minitab SB delivery approach versus traditional vegetarian capsules (9±9 vs. 85±63 au; *p*=0.003; ES=1.62). These novel approaches have not yet been applied to a recovery context; therefore, further research studies are needed before conclusions can be made regarding their efficacy.

Researchers examining the effect of SB ingestion on repeated bout exercise performance have traditionally opted to give the supplement as a single dose ~60–90 min prior to an initial exercise bout, irrespective of whether they use solution or capsule SB administration approaches (Pierce et al. 1992; Zabala et al. 2008, 2011; Mero et al. 2013). Similarities in supplement timing can be traced back to early work examining the effect of SB on blood pH and [HCO_3_^−^] recovery (Costill et al. 1984; Katz et al. 1984). Interestingly though, studies profiling interindividual differences in blood pH and [HCO_3_^−^] responses after 0.3 g kg^−1^ BM SB suggest that peak changes occur ~60–90 min when adopting a solution approach (Gough et al. 2017b; Deb et al. 2018) and between 120 and 240 min when SB is consumed via capsules (Jones et al. 2016; de Oliveira et al. 2020). Given this large degree of variability for HCO_3_^−^ absorption, aligning SB ingestion with pre-determined individualised time-to-peak [HCO_3_^−^] has been recommended (Boegman et al. 2020; Gurton et al. 2021b; Gough et al. 2021). In a series of investigations, Gough et al. (2018, 2019a) studied the effect of individualised SB ingestion on blood buffering capacity during cycling TT’s. Their preliminary study showed that SB increased blood pH and [HCO_3_^−^] compared with a placebo after 40-min recovery from a 4-km cycling TT in hypoxia (~0.08 au and 7 mmol L^−1^, respectively; *p*<0.05). In their follow-up study, Gough et al. (2019a) demonstrated that SB accelerated the restoration of [HCO_3_^−^] between 2 × 4-km cycling TT’s (6.1 mmol L^−1^ higher than placebo; *p*<0.001) separated by 40 min, with elevated blood buffering capacity likely contributing to improved performance during a second 4-km TT. Despite these promising findings, it must be considered that not all athletes have access to blood gas analysis (i.e., financial and/or logistical restraints), and therefore, alternative supplementation timing approaches potentially able to maximise blood buffering capacity during repeated bout exercise events should also be investigated.

SB ingestion has also been administered using a split-dose strategy, including early research by Pruscino et al. (2008) reporting higher [HCO_3_^−^] compared to the placebo at the end of a 30-min recovery period between repeated 200-m swimming TTs (~7 mmol L^−1^; *p*<0.05). In a study examining the effect of SB on repeated “all-out” cycling performance across a single day, Dalle et al. (2019) divided SB supplementation into three identical 3 h cycles: a 6.3 g bolus at the start, followed by 2.1 g doses at +1 h and +2 h, which equated to 0.4 g kg^−1^ BM SB being consumed over 9 h. Importantly, these authors observed elevated blood pH and [HCO_3_^−^] at the end of a 9-h competition day (pH: ~7.47 au; HCO_3_^−^: ~31 mmol L^−1^). The concept of “topping up” with smaller SB doses raised by Dalle et al. (2019) could have important practical implications for athletes performing multiple exercise events separated by shorter recovery durations (e.g., ~60–90 min between track cycling heats and final). It is logical to assume that ~0.2–0.3 g kg^−1^ BM SB ingested pre-exercise combined with a “top up” dose of ~0.1 g kg^−1^ BM SB immediately at the start of recovery will lead to more pronounced changes in blood pH and [HCO_3_^−^] prior to subsequent exercise (compared with only a single SB dose consumed before the initial exercise bout). On the other hand, athletes could consume these small “top up” doses during exercise to replenish [HCO_3_^−^] throughout a competition event (Dalle et al. 2021). Dose–response blood acid–base balance pharmacokinetics data indicate that ~2–3 mmol L^−1^ changes in [HCO_3_^−^] occur after ~40 min for 0.1 g kg^−1^ BM SB (Jones et al. 2016), which suggests that the use of “top up” SB doses during a competition event would likely be limited to endurance exercise (e.g., Grand Tour cycling stages, ~3–4 h) as sufficient time is needed for changes in [HCO_3_^−^] to occur.

Another timing strategy that has emerged within scientific literature is post-exercise SB ingestion (Gough et al. 2017a, 2019a, b; Gurton et al. 2021a). This theoretically ensures that enhanced HCO_3_^−^ buffering is present throughout recovery, as opposed to the initial exercise bout, thus maximising any benefits during subsequent exercise bouts. From a practical perspective, post-exercise SB timing could be of interest to athletes performing multiple events/training sessions on the same competition day, for instance when a heat and/or qualification round is followed by a final of greater importance. During their initial study, Gough et al. (2017a) showed that post-exercise SB elevated blood pH and [HCO_3_^−^] (~0.10 au, 7.0 mmol L^−1^; *p*<0.001) compared with placebo at the end of a 90 min recovery period between two TTE cycling tasks. However, as the authors waited until 30 min post-exercise to administer SB, blood pH and [HCO_3_^−^] had recovered close to baseline prior to supplementation for both conditions, meaning that it was difficult to draw conclusions for the efficacy of post-exercise SB at accelerating blood acid–base balance recovery. In a follow-up study, Gough et al. (2019b) reported elevated [HCO_3_^−^] (8.0±2.1 mmol L^−1^ higher than placebo) after 75-min recovery when SB was ingested 10 min post-exercise, at which point blood [HCO_3_^−^] was still considerably below baseline levels. Importantly, both studies (Gough et al. 2017a; 2019a, b) showed ergogenic effects in the subsequent bout of exercise (100% TTE and a boxing simulation, respectively), suggesting that post-exercise SB ingestion might be an effective timing strategy when sufficient recovery is available. In contrast, post-exercise SB ingestion may not be warranted when only a short recovery period separates exercise bouts. Gurton et al. (2021a) found that SB ingested immediately following TTE running at a velocity corresponding to maximal oxygen consumption (100% v-VO_2max_) did not increase blood pH or [HCO_3_^−^] at 35-min recovery. These authors concluded that insufficient time was available between consuming SB and commencing the second bout of exercise for enough HCO_3_^−^ to be absorbed that would elicit substantial changes in blood acid–base balance, with significant elevations in [HCO_3_^−^] only reported after the second TTE bout (~2.5 mmol L^−1^ higher than placebo). Based on the existing evidence, it seems that when ~75 min recovery is available, post-exercise SB ingestion might be an effective strategy for improving subsequent exercise performance.

## Repeated bout exercise performance

This section of the review focuses on studies that have investigated the effect of SB on performance outcomes during two or more bouts of high-intensity exercise performed on the same day interspersed by ≥10 min. Recent meta-analyses and systematic reviews discuss ergogenic benefits of SB during repeated and intermittent sprint exercise comprising shorter work/rest ratios (Lopes-Silva et al. 2019; Grgic 2022). An overview of methodological design (i.e., dosage, ingestion strategy, and timing) and performance outcomes from studies examining the effect of SB on repeated bout exercise is displayed in Table 1.

**Table 1:** Effect of SB supplementation on repeated bout exercise performance

Study	Participants	Experimental design	Supplementation (dosage, timing & ingestion strategy)	Exercise protocol & recovery duration	Performance outcomes
Pierce et al. (1992)	University male swimmers (n = 7, age = 19.3 ± 0.42 years, BM = 71.6 ± 1.8 kg)	Randomised, double-blind, placebo-controlled, crossover; 3 day washout	SB: 0.2 g kg^−1^ BM; PLA: 1 g NaCl; CON: no drink; single dose, 60 min pre-exercise, 400 ml water	2 × 200-yard swim (style of their choice) separated by 20 min	100 yard time: ↔ 2 × 200 yards: ↔
Pruscino et al. (2008)	Elite male freestyle swimmers (n = 6)	Randomised, double-blind, placebo-controlled, crossover; ≥3 day washout	SB: 0.3 g kg^−1^ BM; PLA: glucose; split into 7 doses, 120–30 min pre-exercise, given in capsules with 20 ml kg^−1^ BM water	2 × 200 m swim freestyle TT separated by 30 min	TT1: ↔ TT2 vs. TT1: ↑
Zabala et al. (2008)	Elite male BMX athletes (n = 9, age = 19.4 ± 2.3 years, BM = 73.8 ± 9.9 kg)	Randomised, double-blind, placebo-controlled, crossover; 4 day washout	SB: 0.3 g kg^−1^ BM; PLA: 0.05 g kg^−1^ NaCl; single dose, 90 min pre-exercise, 1000 ml flavoured water	3 × 30 s WT separated by 30 min; CMJ after WT	Jump height: ↔ WT peak power, mean power and fatigue index: ↔
Zabala et al. (2011)	Elite male BMX athletes (n = 10, age = 20.7 ± 1.4 years, BM = 77.9 ± 2.1 kg)	Randomised, double-blind, placebo-controlled, counterbalanced crossover; 4 day washout	SB: 0.3 g kg^−1^ BM; PLA: matched capsules; single dose, 90 min pre-exercise, given in capsules with water provided ad libitum	3 × 30 s WT separated by 15 min; CMJ after WT	Jump height: ↔ WT peak power, mean power and fatigue index: ↔
Mero et al. (2013)	Elite male swimmers (n = 13, age = 20.5 ± 1.4 years, BM = 80.1 ± 8.1 kg)	Randomised, double-blind, placebo-controlled, crossover; 7 day washout	SB: 0.3 g kg^−1^ BM; PLA: calcium carbonate; single dose, 60 min pre-exercise, given in capsules	2 × 100 m swim freestyle TT separated by 12 min	TT1: ↔ TT2 vs. TT1: ↑
Stöggl et al. (2014)	Endurance trained males (n = 12, age = 32.8 ± 3.8 years, BM = 74 ± 6 kg)	Randomised, double-blind, placebo-controlled, counterbalanced crossover; 5–8 day washout	SB: 0.3 g kg^−1^ BM; PLA: artificial sweetener and NaCl; single dose, 90 min pre-exercise, 6 ml kg^−1^ BM water	3 × TTE at 19 km h^−1^ (5%) separated by 25 min	TTE1: ↔ TTE2: ↑ Decline from TTE1 to TTE2 and TTE3: ↑
Gough et al. (2017a, b)	Recreationally active males (n = 9, age = 23 ± 2 years, BM = 74 ± 9 kg)	Double-blind, placebo-controlled, crossover; 3–10 day washout	SB: 0.3 g kg^−1^ BM; PLA: 0.1 g kg^−1^ NaCl; single dose, 30 min post-exercise, 5 ml kg^−1^ BM water/squash	2 × TTE cycling at 100% *W*_peak_ separated by 90 min	TTE1: ↔ TTE2: ↑
Dalle et al. (2019)	Recreationally active males (n = 12, age = 21 ± 1 years, BM = 74.4 ± 9.9 kg)	Randomised, double-blind, placebo-controlled, crossover; 7 day washout	SB: 31.5 g; PLA: matched NaCl; split over 9 h, every 3 h: 6.3 g + 2 × 2.1 g, given in capsules with 250 ml water	4 × 2 min “all-out” sprints separated by 180 min	Sprint 1, 2 & 4: ↔ Sprint 3: ↑ Average mean power output: ↑
Gough et al. (2018)	Trained male cyclists (n = 10, age = 27 ± 8 years, BM = 82 ± 9 kg)	Randomised, double-blind, placebo-controlled, crossover; 7 day washout	SB: 0.2 g kg^−1^ and 0.3 g kg^−1^ BM; PLA: 0.07 g kg^−1^ NaCl; single dose, time-to-peak bicarbonate pre-exercise, dissolved in 450 ml water/squash	2 × 4 km cycling TT separated by 40 min in hypoxia (FiO_2_ 14.5%)	TT1 and TT2: ↑ Decline from TT1 to TT2: ↔
Gough et al. (2019a, b)	Elite male boxers (n = 7, age = 27.1 ± 5.1 years, BM = 72.2 ± 10.3 kg)	Randomised, double-blind, placebo-controlled, crossover; 7 day washout	SB: 0.3 g kg^−1^ BM; PLA: 0.1 g kg^−1^ NaCl; single dose, 10 min post-exercise, dissolved in 5 ml kg^−1^ BM water/squash	4 × 30 s bouts of 90% and 75% v-VO_2max_ running, boxing specific drills, 2 × TTE runs at 90% v-VO_2max_ separated by 75 min	TTE1: ↔ TTE2: ↑ Change from TTE1 to TTE2: ↑
Peinado et al. (2019)	Elite male BMX athletes (n = 12, age = 19.2 ± 3.4 years, BM = 72.4 ± 8.4 kg)	Randomised, double-blind, placebo-controlled, counterbalanced crossover; 4 day washout	SB: 0.3 g kg^−1^ BM; PLA: 0.045 g kg^−1^ NaCl; single dose, 90 min pre-exercise, given in capsules with water provided ad libitum	3 × BMX races on an outdoor track (400 m length)	Time, peak velocity and time-to-peak velocity: ↔
Gurton et al. (2021a)	Recreational male runners (n = 11, age = 31.0 ± 9.7 years, BM = 74.4 ± 6.5 kg)	Randomised, single-blind, placebo-controlled, crossover; 5–7 day washout	SB: 0.3 g kg^−1^ BM; PLA: 0.03 g kg^−1^ NaCl; single dose, 5 min post-exercise, dissolved in 500 ml water/squash	2 × TTE runs at v-VO_2max_ (1%) separated by 40 min	TTE1: ↔ TTE2: ↔ 6/11 improved above test re-test: ↑
Thomas et al. (2022)	Elite track cyclists (men, n = 6, age = 19.8 ± 1.5 years, BM = 83 ± 6 kg; women, n = 2 age = 21.5 ± 2.1 years, BM = 60 ± 0 kg)	Randomised, double-blind, placebo-controlled, crossover; 7 day washout	SB: 0.3 g kg^−1^ BM; PLA: 0.2 g kg^−1^ BM calcium carbonate; single dose, 90 min pre-exercise, given in capsules with water provided ad libitum	4 × 1000 m constant power efforts separated by 20 min 3 × 500 m “all-out” sprints separated by 20 min, squat jumps pre- and post-sprints	4 × 1000 m constant power efforts: ↔ Average velocity during “all-out” sprints: ↔ Pre-exercise jump height: ↔ Post-exercise jump height: ↑
Thomas et al. (2023)	Active men (n = 8, age = 22 ± 4 years, BM = 80.3 ± 13.0 kg)	Randomised, double-blind, placebo-controlled, crossover; 7 day washout	SB: 0.3 g kg^−1^ BM; placebo: 0.2 g kg^−1^ BM calcium carbonate; single dose, 90 min pre-exercise, given in capsules with water provided ad libitum	3 × 30 s WT separated by 20 min	WT peak power, mean power and fatigue index: ↔

*BM* body mass, *SB* sodium bicarbonate, *PLA* placebo, *NaCl* sodium chloride, *WU* warm up, *WT* Wingate test, *CMJ* countermovement jump, *TT* time-trial, *TTE* time-to-exhaustion, *W*_*peak*_ peak aerobic power output, *v-VO*_*2max*_ maximal aerobic velocity, *VO*_*2max*_ maximal oxygen consumption

↑ Significant improvement (*p* < 0.05), ↔ no significant change (*p* > 0.05)

Some studies have examined the effect of SB on repeated swimming TT performance separated by <30-min recovery (Pierce et al. 1992; Pruscino et al. 2008; Mero et al. 2013). Since athletes competing in elite swimming events sometimes have to perform multiple races for different disciplines during a single competition day (Capelli et al. 1998; Mero et al. 2013), it is possible that if SB can accelerate recovery after an initial event and improve repeated bout exercise performance, then it could be the difference between winning and finishing outside of medal positions. Pierce et al. (1992) reported that SB ingestion had no effect on performance during 2 × 200-yard swimming TTs separated by 20 min recovery. Whilst these authors were unable to measure blood acid–base balance, it is reasonable to suggest that their 0.2 g kg^−1^ BM SB dose administered at a standardised time frame did not adequately increase [HCO_3_^−^] to improve repeated swim performance. In a study examining 2 × 200-m freestyle TTs separated by 30 min, Pruscino et al. (2008) demonstrated a beneficial effect of SB on changes in completion times from TT1 to TT2 (0.7% less drop-off compared with placebo, *p*=0.05). Mero et al. (2013) also reported that SB reduced the decline in performance during 2 × 100-m freestyle swimming TTs separated by 12 min (1.5 s less than placebo, *p*<0.05). Discrepancies in these results for the efficacy of SB during repeated bout swimming TTs could relate to factors, such as participant training status, exercise duration, and/or SB ingestion timing. In particular, ergogenic effects of SB might be greater during middle-distance compared with short-distance swimming events (Grgic and Mikulic 2021), and therefore, future research should explore the efficacy of individualised SB supplementation for improving performance during repeated 200-m or 400-m swimming TT’s.

Accelerating recovery following an initial exercise bout could also have important applied benefits to competitive track cyclists, where repeated high-intensity efforts are a common requirement of training and/or competition (Monedero and Donne 2000; Al-Nawaiseh et al. 2016). For instance, the first round and final of the men’s team pursuit at the Rio 2016 Olympics were separated by ~60 min, meaning that athletes needed to be able to recover quickly between bouts. Two studies have investigated the effect of SB ingestion on repeated cycling TT performance (Gough et al. 2018; Thomas et al. 2022). Firstly, Gough et al. (2018) demonstrated that individualised SB in doses of both 0.2 g kg^−1^ and 0.3 kg^−1^ BM improved 2 × 4-km cycling TT performance in hypoxic conditions compared to a placebo (40-min recovery; TT1/TT2, both ~1.5% faster; *p*<0.05), with minimal differences between doses. More recently, Thomas et al. (2022) reported that SB did not improve performance during 4 × 1-km constant power TT’s separated by 20 min. They did, however, observe lower RPE after the 4 × 1-km cycling TT’s for SB compared to placebo (Borg-CR10: ~2 au less; *p*<0.05). A body of literature exists linking attenuated RPE after SB ingestion to centrally mediated mechanisms (Swank and Robertson 1989; Robertson et al. 1992). This has been underpinned by the assumption that excessive accumulation of H^+^ within intracellular compartments negatively influences force generating capabilities of muscles (Fitts 2016), which is characterised by localized pain sensations (Robertson et al. 1992). SB ingestion leads to peripheral alterations (i.e. fewer intracellular H^+^) that modulate activation of group III and IV muscle afferents, in turn reducing negative feedback from muscles and sustaining drive to motor neurons that lowers RPE (Amann et al. 2015). Importantly, from a practical sense, reducing RPE throughout near maximal intensity events could have notable real-world implications for athletes competing in multiple competitions on the same day.

A number of studies have examined the effect of SB during repeated “all-out” cycling exercise, most likely as BMX events require athletes to repeatedly perform high-intensity efforts during qualification phases compromising at least three rounds (~30 min recovery between each) (Peinado et al. 2019). In a series of investigations from Zabala et al. (2008, 2011) and Thomas et al. (2023), it was found that SB did not improve performance during 3 × 30-s Wingate cycling tests interspersed by 15–30-min recovery periods. Peinado et al. (2019) observed no ergogenic benefits of SB during three competitive BMX races (~30 s duration) each separated by 15 min. Thomas et al. (2022) also reported no improvements in average velocity for SB during 3 × 500 m “all-out” cycling sprints. These type of exercise tests comprising “all-out” efforts might not be sensitive enough to observe performance benefits after SB (Grgic 2022). Improvements in performance are most likely to be shown when the rate of decrease in intramuscular pH is relatively slow, typically during exercise lasting 4–8 min (Matson and Tran 1993). Rapid rate of decline in pH during “all-out” cycling tests results in the monocarboxylate transporter 1/4 becoming oversaturated with H^+^, as accumulation in intracellular compartments overwhelms clearance rates (Messonnier et al. 2007). This means that the beneficial effects of enhanced circulating HCO_3_^−^ on restoring intracellular acid–base balance are diminished, subsequently limiting the ergogenic capacity of SB (Higgins et al. 2013).

Dalle et al. (2019) showed SB to increase mean power during 4 × 2 min “all-out” cycling tests separated by 180 min throughout an endurance cycling protocol (1.4% higher than a placebo; *p*=0.035). As alluded to, their longer exercise duration is one explanation for why performance benefits were observed for SB. Interestingly, Thomas et al. (2022) reported greater squat jump height for SB after their “all-out” cycling sprints (~2.5% compared with placebo; *p*<0.05). Inconsistencies between the effect of SB ingestion on vertical jump height and “all-out” cycling could be attributed to their differing energy system requirements (Thomas et al. 2022). Short-duration plyometric exercises are predominately influenced by changes in phosphocreatine (PCr) hydrolysis opposed to glycolytic flux (Hultman et al. 1967), which suggests that they might be more sensitive to post-exercise kinetics of PCr recovery. Further mechanistical work is required, but since PCr recovery following high-intensity exercise is somewhat associated with muscle acidity (Lodi et al. 1997), it is possible that elevated acid–base balance after SB ingestion contributes to improvements in post-exercise explosive plyometric movement tasks such as vertical jumps that are reliant on the ATP-PCr system.

Finally, there is a growing body of scientific literature investigating the ergogenic capacity of SB ingestion during repeated TTE bouts (Stöggl et al. 2014; Gough et al. 2017a, 2019b; Gurton et al. 2021a), with equivocal findings to date. Indeed, Stöggl et al. (2014) reported benefits during 3 × 19 km h^−1^ bouts (25 min between each, TTE2: +15% higher for SB than control, *p*=0.048), but Gurton et al. (2021a) observed no differences between two bouts of exhaustive running (100% v-VO_2max_) separated by 40 min. The inconsistency in findings might be due to the timing of SB ingestion, as Gurton et al. (2021a) adopted a post-exercise SB ingestion strategy that may have allowed sufficient time to observe substantial changes in [HCO_3_^−^] that would lead to ergogenic benefits during subsequent exercise. In support of this idea, Gough et al. (2017a) reported that administering SB 30 min after an initial TTE cycling bout improved performance during a second TTE cycling test after a longer, 90-min recovery (+16.6% greater than placebo; *p*=0.007). However, as blood pH and [HCO_3_^−^] prior to the second TTE task were reflective of studies opting for pre-exercise SB ingestion, it should be noted that their results do not directly suggest enhanced recovery per se, but instead perhaps show the known ergogenic effects of pre-exercise SB. In their follow-up study, Gough et al. (2019b) demonstrated that change in performance from TTE1 to TTE2 (separated by 75 min) was greater when SB was administered 10 min after the initial exercise (91 s longer TTE than placebo; *p*=0.02). Based on this evidence, the effects of SB ingestion on repeated TTE performance are promising, although as athletes would rarely complete two exhaustive tasks to exhaustion, the practical application of this is unclear.

## Practical implications and the sodium bicarbonate ingestion recovery framework

Based on the findings of this narrative review Table 2 depicts an SB ingestion recovery framework. It is envisaged that this SB ingestion recovery framework will act as a tool for practitioners and athletes wishing to easily identify where ingestion may aid exercise performance across repeated bouts of exercise. In short, it is proposed that scenarios where only a recovery duration of up to 30 min exists between bouts of exercise, SB ingestion is unlikely to provide ergogenic benefits to performance. On the other hand, between 30 and 90 min of recovery seems to be the optimal for eliciting ergogenic benefits for SB, as there is sufficient acid base balance recovery between each bout, which then can contribute to improvements during the subsequent bout of exercise. To date, no research has explored the effect of a single SB dose on performance in two or more exercise bouts separated by more than 90 min, and thus, further studies examining this time frame are required to refine practical recommendations. Finally, the concept of ‘top-up’ SB doses is a novel area that has shown promise and could be particularly beneficial for athletes competing in endurance exercise.

**Table 2:** Practical framework for the ingestion of sodium bicarbonate during repeated bout effort sporting events

Recovery time frame between exercise bouts	Recommendation	Recommended scenarios for use	Sports that could benefit	References	Future research directions
0–30 min	SB unlikely to be effective as acid base balance cannot recover in time, although some benefits have been observed with three bouts of exercise (*)	During half-time to improve second half performance Qualification rounds separated by a short rest period	Team sports (football/soccer, rugby) BMX cycling	Pruscino et al. (2008), Mero et al. (2013), Pierce et al. (1992), Thomas et al. (2022), Zabala et al. (2008), Peinado et al. (2019), Thomas et al. (2023)	Individualised time-to-peak alkalosis ingestion strategy may provide more consistent benefits and a stronger recovery profile of acid–base balance
30–90 min	0.2–0.3 g kg^−1^ BM SB either prior to or post the initial bout of exercise *standardised or individual time-to-peak ingestion timing* (***)	Heat and final events whereby only a short recovery is available Training whereby repeated bouts are completed within a short time frame	Cycling (primarily track), swimming, sprint skiing	Gough et al. (2018), Gough et al. (2017a, b), Gough et al. (2019a), Gough et al. (2019b), Gurton et al. (2021a, b)	Pre- versus post-exercise ingestion of SB Investigations into ecologically valid sporting scenarios
90+ min	Promise for SB to work, but research lacking (*)	Heat and final events whereby a longer recovery is available Training whereby repeated bouts are completed on the same day	Some swimming events, rugby 7’s	N/A	Studies examining longer time frames of recovery are required as none to date have gone past 90 min recovery for a single SB dose
During exercise/a competition event	Initial and “top-up” doses may be effective for endurance-based exercise, with SB ingestion also prior to exercise (**)	Grand Tour stage races (3–4 h). Use a pre-exercise dose and then top-up halfway through	Endurance cycling or running events	Dalle et al. (2019), Dalle et al. (2021)	Due to minimal scientific evidence, further studies investigating a range of sports are required

The table has been constructed based on the evidence in the area and the expertise within the authorship group in both research and applied experiences with an end goal to be used a practical point of reference for practitioners and athletes within their given sports

*BM* body mass; *SB* sodium bicarbonate

Symbols denote level of empirical evidence for the efficacy of SB: * little-to-none despite extensive research, or more research required before conclusions can be made, ** might be beneficial in specific circumstances, *** high likelihood of performance benefits

## Future research directions

Since existing literature has mainly focused on the pre-exercise effects of SB ingestion, it is recommended that greater attention is given to the application of SB as a recovery aid in the future (using either pre- or post-exercise ingestion). First, from a mechanistical viewpoint, further research is needed examining the influence of SB ingestion on recovery of the three pertinent physiological systems/pathways discussed in this review: (1) acid–base balance, (2) SID, and (3) cellular stress responses and oxidative stress. Particular focus should be given to whether SB mediated changes in cellular stress responses (e.g., HSP72, oxidative stress) lead to noticeable improvements in recovery from exercise, repeated bout performance, and training adaptations. Future mechanistical work could also examine the role of SB on inorganic phosphate accumulation and calcium signalling during repeated bout exercise, as these contribute to skeletal muscle contractile function (Allen and Trajanovska 2012). Second, it would be worthwhile to examine the importance of SB timing by comparing the effect of pre- and post-exercise SB ingestion on blood pH, [HCO_3_^−^] and strong ions across different recovery durations. In a similar fashion, exploration into the concept of “top up” SB doses would be beneficial based on promising findings of Dalle et al. (2019) during endurance exercise. The use of SB is not typically associated with endurance exercise, yet these “top up” doses could be useful for athletes competing in longer duration sports (e.g., Grand Tour cyclists). Additionally, research is warranted comparing factors such as sex-specific or training status differences that may influence the efficacy of SB as a recovery aid, as this remains an underexplored area that could have important real-world implications for practitioners and athletes.

Finally, since studies discussed in this review have primarily concentrated on cycling and swimming, it is important for future research to investigate other exercise modalities and events that could potentially benefit from accelerated indices of recovery between repeated bouts of exercise. One example could be during 15–20 min half-time periods in team sports (e.g., rugby, soccer, and basketball), as existing evidence suggests that SB ingestion increases blood pH, [HCO_3_^−^] and SID during similar recovery durations (Verbitsky et al. 1997; Siegler et al. 2008). Other sporting disciplines that could be of interest to the application of SB as a recovery aid are rugby sevens and cross-country skiing, as they feature excessive H^+^ accumulation and short recovery period between exercise bouts (Ross et al. 2014; Losnegard 2019).

## Conclusion

Based on the literature, it is reasonable to suggest that SB ingestion could play a key role in improving recovery of acid–base balance and thus performance, given the correct ingestion strategy and recovery period between two bouts of exercise. In light of this, the ingestion framework presented within this review may serve as a tool for practitioners to use with athletes. Further research is needed to determine the optimal SB ingestion approach for athletes in multiple sports to further explore the role of this supplement on sports performance.

## Data Availability

Data collected from literature search is available upon reasonable request.

## Abbreviations

ATP: Adenosine triphosphate
au: Arbitrary units
BM: Body mass
Cl^−^: Chloride ion
GI: Gastrointestinal
GSSG: Oxidised glutathione
H^+^: Hydrogen ion
HCO_3_^−^: Blood bicarbonate
HSP72: Heat shock protein 72
K^+^: Potassium ion
Na^+^: Sodium ion
PCr: Phosphocreatine
ROS: Reactive oxygen species
SB: Sodium bicarbonate
SID: Strong ion difference
TBARS: Thiobarbituric acid reactive substances
TGSH: Total glutathione
TT: Time-trial
TTE: Time-to-exhaustion
v-VO_2max_: Running velocity at maximal oxygen consumption
